# The effectiveness and limitation of the national childhood hepatitis A vaccination program in the Republic of Korea: Findings from the Korean National Health and Nutrition Examination Survey (KNHANES), 2015

**DOI:** 10.1371/journal.pone.0189210

**Published:** 2017-12-08

**Authors:** Juwon Lim, Kyuwoong Kim, Seulggie Choi, Sang Min Park

**Affiliations:** 1 Department of Medicine, Seoul National University, Seoul, Korea; 2 International Healthcare Center, Seoul National University Hospital, Seoul National University College of Medicine, Seoul, Korea; 3 Department of Biomedical Sciences, Seoul National University Graduate School, Seoul, Korea; 4 Department of Family Medicine, Seoul National University Hospital, Seoul National University College of Medicine, Seoul, Korea; University of North Carolina at Chapel Hill School of Medicine, UNITED STATES

## Abstract

**Background:**

Vaccination for hepatitis A virus (HAV) has been implemented as one of the national vaccination programs despite the epidemiological transition of HAV in the Republic of Korea. While the national HAV vaccination program is largely associated with the shift of socioeconomic trend in the country, concerns have been raised on the effectiveness of the HAV immunization. The objective of this study was to examine the epidemiological trend of HAV and assess the effectiveness of the nationwide HAV vaccination policy based on a nationally representative sample of the Korean population collected in 2015.

**Methods:**

We analyzed anti-HAV of 5,856 respondents aged ≥10 years collected from Korean National Health and Nutrition Examination Survey (KNHANES) data in 2015. We estimated age-adjusted anti-HAV prevalence by sociodemographic and other characteristics. We evaluated the factors associated with anti-HAV positivity among each age group (10–19, 20–29, 30–45 and over 45 years old).

**Results:**

The prevalence of anti-HAV among adults aged ≥10 years was 72.5% (95% confidence interval, CI, 73.7–71.4) in 2015. The lowest age-specific prevalence was among adults aged 20–29 years with 11.9% (95% CI 9.3–15.1%). The prevalence of anti-HAV among those aged 10–14 and 15–19 years was 59.7% (95% CI 52.7–66.4) and 24.0% (95% CI 19.5–29.3), respectively. The prevalence of anti-HAV among adults aged between 30 and 44 years rapidly increased from below 20% to above 90%. The prevalence of anti-HAV among adults aged ≥45 years was 97.8% (95% CI 96.0–97.6). Factors significantly associated with anti-HAV positivity among those aged 10–19 years old were young age, higher house income and high influenza vaccination rate. Compared to the respondents aged 10–19 years (those who were subject to the national childhood vaccine recommendation), those aged 20–29 years (those who were not subject to the recommendation) had low adjusted odds ratio (OR, 0.52 95% CI 0.34–.81 P-value = 0.004) for anti-HAV positivity.

**Conclusions:**

The age-adjusted anti-HAV prevalence showed a U-shaped association, implying the high dependence of anti-HAV prevalence on age and the epidemiological shift. The inclusion of the hepatitis A vaccine into the national immunization recommendation was effective shown by the increase of immunity in the general population. However, the vaccination rate was low in the low-income group. Young adults aged 20–39 years may benefit from inclusion in the HAV vaccination program due to the significantly low vaccination rate.

## Introduction

Hepatitis A virus (HAV) is an RNA virus [1] transmitted by the fecal-oral route, either by person-to-person contact or by ingestion of contaminated food or water [2, 3]. The global burden of HAV disease is high with an estimated 1.4 million cases and over 27,731 associated deaths in 2010[4]. Patterns of this disease vary geographically into three or four general groups [5, 6]. Most Latin American, Asian, and Middle Eastern nations with high levels of infection among young children have few outbreaks of HAV infection since immunity is acquired at an early age when infection is commonly asymptomatic. In countries such as Japan, South Korea, and Singapore with intermediate levels of infection, improved sanitary conditions, water quality, and hygiene leads to decreased exposure among children, but also increased children and adults who may be susceptible. Countries such as Australia, New Zealand, Canada, the United States, and most European nations with low and very low rates of infection in all age groups have infrequent outbreaks of HAV in any age group. Therefore, the socioeconomic developmental patterns of nations can alter the infection rate over time [6, 7].

Recently, some nations have seen a new pattern of hepatitis A outbreaks among young adults [6, 8–10]. This phenomenon is likely due to the lack of immunity of young adults, who were not vaccinated for HAV and not exposed to HAV as a child. Therefore, young adults remain susceptible to hepatitis A due to this lack of immunity. This is particularly problematic as the clinical course of HAV infection is more severe with increasing age and may even be fatal [11, 12].

South Korea, as well as other countries, have noticed this new pattern of HAV infection recently [13–15]. During the past decade, the level of HAV infection changed from intermediate to low levels, due to the elevated sanitary conditions, socioeconomic development, and national childhood vaccine programs. Particularly, universal vaccination for HAV was recommended for all children aged over 12 months since 1997 as a part of the national childhood vaccination program in South Korea [16]. The incidence rate of hepatitis A decreased in South Korea until 2011, when the incidence rate was the lowest recorded (0.4 cases per 100,000 population) as a consequence of these changes [17]. However, several nationwide outbreaks of HAV occurred from 2007 to 2011, especially among young adults [11, 18]. The year 2009,in which 15,231 cases of HAV infection were reported, was the largest HAV outbreak in a decade, 87.1% of the patients being adults [19]. This phenomenon is likely due to the gap created between the moment of implementation of the HAV vaccination program and the decrease in the incidence of hepatitis as a result of economic development. After these outbreaks, the government funded free HAV vaccines for all children under 12 years old since 2015. Nevertheless, the possibility of HAV outbreaks are thought to exist among teenagers and young adults aged 20–39 years who were not included in the vaccine program.

The aim of the study was to investigate the epidemiological shift of HAV by measuring the seroprevalence of HAV and figuring out the characteristics associated with HAV positivity based on a nationwide representative data of the general South Korean population.

## Methods

### Survey design and study population

The data used in this study was based on a nationally representative sample from the Korea National Health and Nutritional Examination Survey (KNHANES) 2015, conducted by the Korean Ministry of Health and Welfare. KNHANES is a series of national surveys administered to a sample of the noninstitutionalized civilian population in Korea. The survey includes information on sociodemographic health, and nutritional status via three components: health interview, health examination, and nutrition survey. The design and methods of KHNANES are described in detail elsewhere [20]. The institutional review board (IRB) of the Korean Centers for Disease Control reviewed and approves the KNHANES survey annually (KNHANES 2014 IRB approval no.: 2013-12EXP-03-5C). IRB approval was not required for KNHANES 2015 because this survey was conducted for the purpose of public welfare. We were thus exempt from obtaining additional ethical approval for this study. Our study included 5,856 respondents aged ≥10 years.

### Laboratory testing for anti-HAV

Serum specimens from persons aged ≥10 years were tested for HAV antibody using Chemiluminescent Micro particle Immunoassay (ARCHITECT Anti-HAV, ARCHITECT i4000Sr (ABBOTT/Germany). Initial positive results were confirmed with a second HAV antibody testing. Presence of anti-HAV indicates immunity against HAV infection acquired either from past infection or vaccination.

### Definitions and measures

To identify current positivity of anti-HAV, we used baseline characteristic variables including sex, age, and residential area which were obtained by a self-reported survey from all KNHANES participants. Age was categorized into 10–14, 15–19, 20–29, 30–45, and >45 years. Residential area was divided into rural or city.

For analysis of factors associated with HAV immunity among those aged 10–19 years, we selected age, gender, residential area, corrected house income (annual house income divided by square root of the number of family members), parents’ education levels, and annual influenza vaccination as covariates. The quartile categories were used regarding corrected annual house income. Parents’ education status was categorized into three levels: both ≤high school, one ≤high school and the other ≥college, and both ≥college. Annual vaccination of influenza was defined as a self-reported answer to the question “Have you ever received a seasonal influenza vaccination during the last 1 year?”

**Fig 1:**
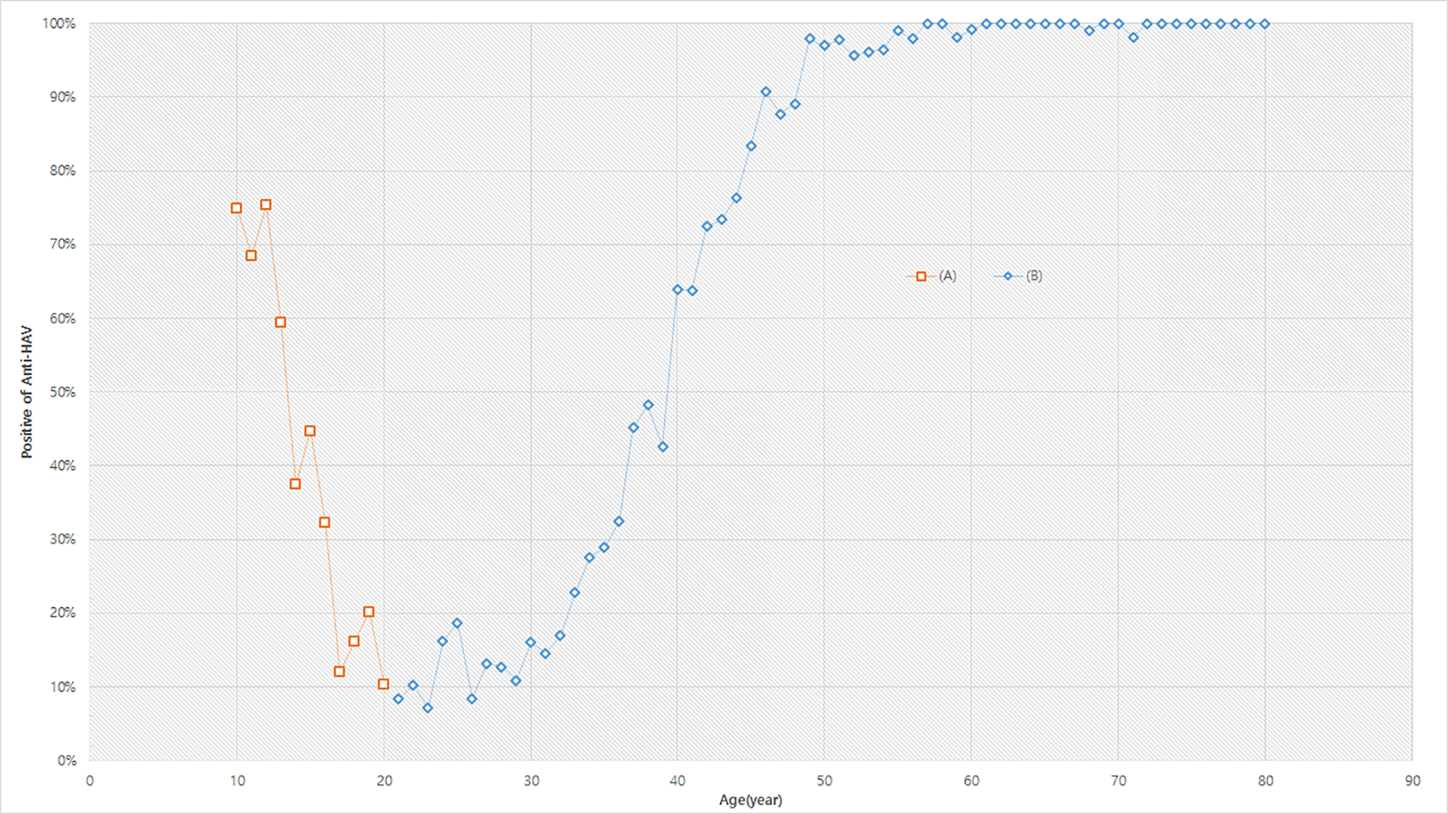
Age-specific prevalence of anti-HAV, persons aged ≥10 years, KNHANES 2015. (A) Included in the national immunization recommendation of hepatitis A vaccine (B) Not included. In South Korea, the HAV vaccine for the children aged 12–26 months started in 1997 as one of the National Childhood Vaccine Program. Children aged 10–19 years in 2015 were recommended for HAV vaccine, but aged 20 years and over were not recommended.

For analysis of the associated factors with the anti-HAV antibody positivity among each age-group, we divided the participants into those aged 20–29, 30–44 and over 45 years according to the new pattern of the positivity of anti-HAV (Fig 1). Covariates included age, gender, residential area, corrected house income, and participant education level. The education status was categorized into two levels: ≤high school, and ≥college.

### Statistical analysis

Estimates were weighted to represent the total non-institutionalized South Korean population and to account for sampling methods response rate.

We estimated anti-HAV prevalence and 95% confidential intervals (Cis) by age-groups, gender, residential area, corrected house income, parents’ education level, and annual influenza vaccination. We used Chi-square tests for statistical comparisons among these subgroups for estimated weighted prevalence.

Persons who were aged 10–19, 20–29, 30–44 and over 45 years were analyzed separately to assess the factors associated with anti-HAV antibody positivity. In analyses for factors associated with anti-HAV positivity, crude prevalence ratios were obtained using a separate logistic regression model for each of the independent variables. All variables that were in simple logistic models were included in the initial multivariate modeling.

A p-value <05 was considered significant for all tests. STATA (version 14.2), a statistical package designed to analyze complex survey data, was used for all analyses conducted in this study.

## Results

A total 5,856 participants aged 10 years or more participated in this study. There was a U-shaped association between anti-HAV prevalence and age (Fig 1). The prevalence of anti-HAV among those aged ≥10 years was 72.5% (95% CI 73.7–71.4). The prevalence of anti-HAV among those between the ages 10 and 19 years decreased over in increasing age from above 70% to below 20%. The prevalence of anti-HAV was among adults aged 20–29 years was below 20%. The prevalence of anti-HAV among adults aged between 30 and 45 years rapidly increased with increasing age from below 20% to above 80%. The prevalence of anti-HAV among adults aged ≥50 years was above 90%.

**Fig 2:**
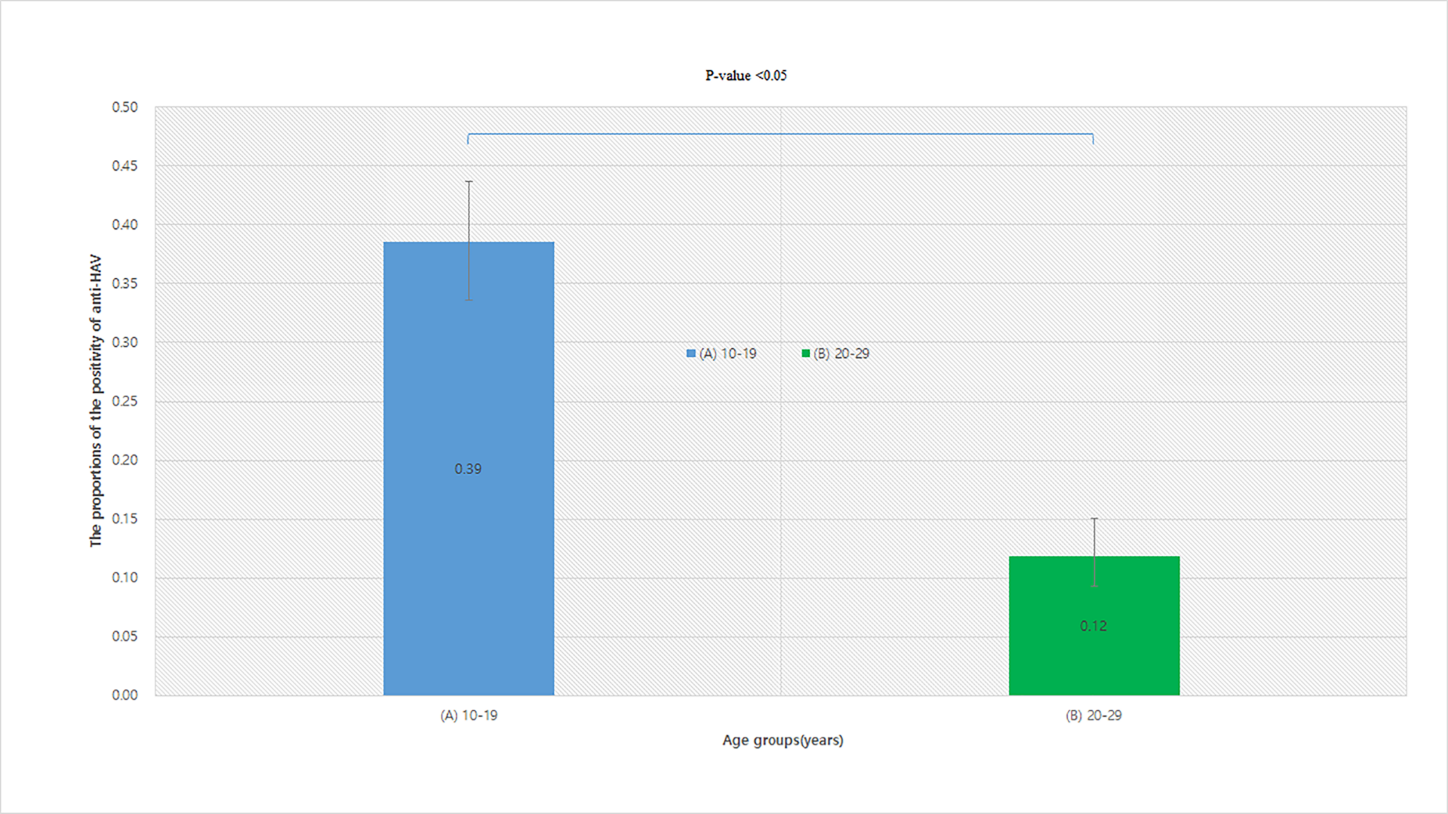
The adjusted proportions of the positivity of anti-HAV among those who were and were not subject to the childhood vaccine program. (A) Included in the national immunization recommendation of hepatitis A vaccine (B) Not included. In South Korea, HAV vaccination for children aged 12–26 months started in 1997 as a part of the National childhood Vaccine Program. Children aged 10–19 years in 2015 were recommended for HAV vaccination, but those aged 20–29 years were not.

Fig 2 shows that the adjusted proportions with 95% CI of the positivity of anti-HAV among those who were and were not subject to the National Childhood Vaccine Program were 0.119 (95% CI 0.093–0.151) and 0.385 (95% CI 0.336–0.437), respectvely (P-value<0.05).

Estimated baseline demographic characteristics and the weighted percentages of the positivity of anti-HAV are shown in Table 1. The mean age (standard error) of the participants was 47.4 (0.2) years, and the number (%) of men and women were 3,300 (54.6) and 2,656 (45.4), respectively. There were approximately 4,763 (81.3%) participants residing in cities and 2,195 (37.5%) participants vaccinated for influenza within the last year. Among those aged 10–19 years, 122 (18.2%) participants had both parents who graduated from college, 167 (24.9%) participants with one parent who graduated from college, and 382 (56.9%) participants with both parents who graduated from high school or below. Anti-HAV positivity was significantly high (all p < .05) among those aged 45 years or more and lowest (11.9, 95% CI 9.3–15.1) among for those aged 20–29 years (Table 1). The prevalence of anti-HAV among those aged 10–14 and 15–19 years was 59.7% (95% CI 52.7–66.4) and 24.0% (95% CI 19.5–29.3), respectively. The prevalence of anti-HAV among those aged 20–29, 30–44 and ≥45 years groups were 11.9% (95% CI 9.3–15.1), 44.6% (95% CI 41.9–48.7), and 97.8% (95% CI 96.0–9760), respectively. There were high positivity of anti-HAV rates among women (65.4%), those residing in cities (72.1%), those with the lowest house income (76.1%), those with parents who have high education levels (53.1%), low individual education level (78.4%), and high influenza vaccination rates (77.0%), compared to men (62.6%), those residing in rural areas (62.4%), within 2–4 quartiles of house income (62.1%, 56.5%, 64.0%), low parental education levels (32.4%, 44.0%), high individual education levels (51.5%), and low influenza vaccination rates (56.9%). All p-values among the mentioned these categories were <0.05.

**Table 1:** The characteristics and the weighted prevalence of anti-HAV.

Characteristic	No. of tested	No. of positive	Weighted % positivity of anti-HAV	95% CI ^b^	P-Value^a^
Age, mean(SE)	47.4 (0.2)				
Age group (years)					**<0.001**
10–14	328	207	59.7	52.7–66.4	
15–19	343	87	24.0	19.5–29.3	
20–29	579	67	11.9	9.3–15.1	
30–44	1,210	654	46.6	41.9–48.7	
45+	3,396	3,322	97.8	96.0–97.6	
Sex					**0.025**
Male	2,656	1,894	62.6	60.3–64.8	
Female	3,200	2,353	65.4	63.2–67.5	
Residual Area					**0.005**
Rural	4,763	3,362	62.4	60.4–64.3	
Cities	1,093	885	72.1	65.8–77.6	
Corrected House Income					**<0.001**
1Q^c^	1,401	1,185	76.1	72.2–79.5	
2Q	1,436	1,023	62.1	58.6–65.5	
3Q	1,523	979	56.5	53.2–59.8	
4Q(high)	1,458	1,028	64.0	62.0–56.7	
Parents Education Levels(aged 10–19)					**0.003**
Both ≤high school	382	139	32.4	26.7–38.6	
High school, college	167	88	44	34.3–54.2	
Both ≥college	122	67	53.1	41.7–64.1	
Individual Education Level(aged > = 20)					**<0.001**
≤High school	3,130	2,691	78.4	76.0–80.6	
≥College	1,562	906	51.5	48.0–54.9	
Annual Influenza Vaccination					**<0.001**
Yes	2,195	1,863	77.0	74.2–79.5	
No	3,229	2,052	56.9	54.8–59.0	

Persons aged ≥10 years, KNHANES 2015.

^a^ Chi-square test of difference at each level of age group, sex and residual area.

^b^ CI: Confidential intervals,

^c^ Q: quartiles

Among those aged 10–19 years, factors having a significant association with high anti-HAV positivity in multivariate models were young age, high corrected house income, and high annual influenza vaccine rate. (Table 2). Gender differences were not observed. Compared to those aged 10–14 years, those aged 15–19 years had lower crude OR (0.31, 95% CI 0.24–0.41) and adjusted OR (0.26, 95% CI 0.18–0.37) for anti-HAV positivity, while residual area, and parents’ education levels showed no significant differences. Compared to the lowest quartile group of house income, the highest quartile group had greater anti-HAV positivity (OR 2.98, 95% CI 1.46–6.11; P for trend = 0.002) among adolescents. Compare to the annual vaccine group, the no vaccination group had lower adjusted OR (0.52 95% CI 0.34-.081 P-value = 0.004)

**Table 2:** Factors associated the positivity of anti-HAV among age groups.

Factors	Crude Proportion (%)	Adjusted PR^a^	Crude Proportion (%)	Adjusted PR	Crude Proportion (%)	Adjusted PR	Crude Proportion (%)	Adjusted PR
**Age group (years)**	10–19		20–29		30–44		45+	
**Sex**								
** Male**	55.5	1	0.48	1	0.41	1	42.5	1
** Female**	44.5	1.1 (0.74–1.62)	0.52	1.03 (0.58–1.8)	0.59	0.78 (0.59–1.03)	57.5	1.42 (0.87–2.31)
**Residual Area**								
**Cities**	86.3	1	0.90	1	0.85	1	78.1	1
**Rural**	13.7	0.7 (0.31–1.54)	0.10	1.20 (0.44–3.20)	0.15	1.23 (0.83–1.83)	21.9	1.36 (0.56–3.27)
**Corrected House income**								
**1Q**	17.5	1	0.13	1	0.10	1	33.7	1
**2Q**	24.8	1.45 (0.71–2.94)	0.27	0.89 (0.33–2.36)	0.24	0.96 (0.54–1.70)	24.4	0.47 (0.18–1.23)
**3Q**	31.6	**2.03** **(1.04–3.96)**	0.32	0.66 (0.25–1.46)	0.34	0.81 (0.45–1.46)	20.3	0.8 (0.29–2.25)
**4Q**	26.1	**2.98** **(1.46–6.11)**	0.28	1.05 (0.39–2.83)	0.32	0.97 (0.53–1.77)	21.7	1.06 (0.35–3.21)
**Parents Education level**								
**both ≤high school**	57.5	1	n/a		n/a		n/a	
**high school, college**	25.2	1.15 (0.67–1.97)						
**both ≥college**	17.3	1.4 (0.78–2.51)						
**Individual Education Level**								
**≤high school**			0.53	1	0.36	1	81	1
**≥college**			0.47	1.02 (0.52–2.01)	0.64	0.86 (0.60–1.23)	19	**0.51** **(0.28–0.91)**
**Annual influenza vaccine**								
**Yes**	30.3	1	n/a		n/a		n/a	
**No**	69.7	**0.52** **(0.34–0.81)**						

4 Age groups: 10–19, 20–29, 30–44 and over 45 years. KHNAES 2015

^a^ PR. Prevalence ratio. PR were adjusted by age, sex, residual area, corrected house income, parents education levels and annual influenza vaccine among aged 10–19 years. PRs were adjusted by age, sex, residual area, corrected house income and individual education level among aged 20–29, 30–44 and over 45 years.

Among those aged 20–29 years, there was no significant difference in anti-HAV positivity according to subgroups. Among the those aged 30–45 years, only age was related to the positivity of anti-HAV. Among those aged 45 years or more, age and individual education level was related to high anti-HAV positivity. High individual education participants had low adjusted OR (0.51, 95% CI 0.28–0.91 P-value = 0.023) compared participants with low levels (Table 2).

## Conclusion

### Ongoing of epidemiological shifting in South Korea

The prevalence of individuals with anti-HAV antibody within the South Korean general population in 2015 was 72.5%, which showed U-shaped association with age. In the past decade, HAV was minor problem among adults in South Korea, as nearly all South Koreans acquired HAV antibodies through natural infection during childhood. However, the improvements in the socioeconomic status and general public health of South Korea have led to a gradual decrease of the positive rate of anti-HAV. Furthermore, HAV vaccination became part of the national childhood vaccination program since 1997, leading to increased immunity among children. One study showed that positive rates of anti-HAV among those aged 1–10 and 11–20 years in South Korea were 6.4% and 7.7% in 2008, respectively[21]. In 2010 and 2014, HAV seroprevalence of a Korean pediatric population younger than 10 years has recently been reported to be 55.5% and 67.7%, respectively[13, 22]. The seroprevalence among adolescents aged between 14 and 17 years was 15.4% in 2011 [16]. Our study showed anti-HAV seroprevalence of those aged 10–14 and 15–19 years to be 59.7% and 24.0%, respectively. Because national recommendation of the HAV vaccine started in 1997 among those aged 12–23 months, these people would be aged 19–20 years in 2015.

Because the HAV vaccination was not yet widely used, most individuals over aged 20 years with anti-HAV antibodies acquired immunity through natural infection. The new pattern of epidemiological transition from community acquired to vaccine induced immunity is ongoing in South Korea, and have a potential to become a public health crisis among young adults. In 2010, the positive rate of anti-HAV was only 14.2% among those aged 21–30 years and greater than 90% among those aged over 41 years [22]. In our study, the positivity of anti-HAV among those aged 20–29 years was below 15%, whereas anti-HAV positivity among adults over 50 years was more than 95%. These results suggest that young adults aged 20–29 years are susceptible to hepatitis A due to the low proportion of anti-HAV positive individuals.

### Factors associated with anti-HAV positivity among those aged 10–19, 20–29, 30–44 and 45 years or more

In South Korea, the national HAV vaccine program for children aged 12–26 months started in 1997. The prevalence changes of anti-HAV among aged 10–19 years old were due to vaccine induced immunity. In South Korea, only a small number of children are no infected with HAV [23]. Thus, only few children had the antibody after HAV infection without vaccination. HAV vaccination was recommended as a part of the national childhood vaccine program. However, HAV vaccination was not provided by the government, and therefore the parents were responsible for the cost of vaccine for their children. Therefore, the economic discrepancy of the immunity against HAV among those aged 10–19 years may have occurred as the consequence of this policy. Our results support the possibility that one of the important barriers for HAV vaccination among those aged 10–19 years was income. One study showed that a positive correlation was observed between monthly family income and HAV vaccination rate, as well as the parent’s education level [16]. Furthermore, influenza vaccination was also associated with positivity of anti-HAV. It is likely that health behavior for vaccine use is not only limited to a single disease, but rather a number of disease preventable by vaccination. One study showed that the most significant reason for receiving vaccination was recommendation from health care providers [16]. Therefore, high HAV vaccination rates can be achieved by providing government funds, and the encouragement of doctors to get HAV vaccines. In summary, the impact of the national childhood vaccine program for HAV was effective in increasing the immunity among those aged 10–19 years. However there were discrepancies in anti-HAV positivity within subgroups of house income and annual influenza vaccination. The campaign for childhood universal vaccination over 12 months have continued, and a government-funded nationwide routine vaccination program of children younger than 12 years started in 2015 to prevent hepatitis A outbreak in the future. The effectiveness and limitation of this new 2015 program should be assessed later on.

Compared to teenagers, those aged 20–29 years seem to be most susceptible to hepatitis A outbreaks. In the past, young adults had mostly HAV immunity, and HAV vaccine was not necessary when they go abroad. However, current young adults have a greater chance of being infected with HAV upon travelling to high-prevalence areas of HAV. One of the main reasons for the rapid decrease in seropositivity among those aged 30–44 is the recent economic development in South Korea. Among those aged 45 years and more, participants with low education showed higher positivity compared to those with high education levels, which has also been shown in previous studies. The economic development and HAV vaccination provided by the government was not sufficient in enhancing the immunity among teenagers. This is likely due to the fact that free HAV vaccination for those below 12 years starting from 2015 did not cover this particularly population.

The hepatitis A vaccines are highly effective in preventing hepatitis A in individuals and in reducing disease incidences in communities [24], with reductions of hepatitis A incidence of 90% or greater [25]. Routine hepatitis A vaccination of children in areas with high rates of hepatitis A since 1999 was a cost-effective strategy and dramatically reduced the burden of hepatitis A in the United States [26, 27], Furthermore universal vaccination was cost saving compared with a regional vaccination policy in the United States [28], China [29, 30], and Chile [31].

However, universal HAV vaccine in childhood was not enough in some countries, requiring additional programs and strategies. In Israel, a universal toddlers-only immunization program without catch-up vaccination highlighted the need for a catch-up vaccination program [8, 32]. In 2014, the Advisory Committee on Immunization Practices in the US revealed the low seroprevalence and high susceptibility to hepatitis A among young adults and suggested expanding of vaccine recommendation to adults [33]. In Greece, HAV vaccine was included in the routine national childhood immunization program since 2008 [34]. One study showed that the catch-up hepatitis A vaccination among children could become cost effective at a variable threshold [35]. However, the national hepatitis A immunization program in the targeted population had a significant impact with even relatively modest vaccine coverage in Australia, [36].

Therefore, we suggest that catch-up HAV vaccination directed at individuals aged 10–49 years (or at least among children) should be considered, in addition to the current vaccination program. This strategy would rapidly reduce the disease burden of hepatitis A not only in South Korea and but also in other countries which are experiencing a similar epidemiologic shift to South Korea. However, the assessment the cost-effectiveness of HAV vaccination for children and young adults is necessary.

**Fig 3:**
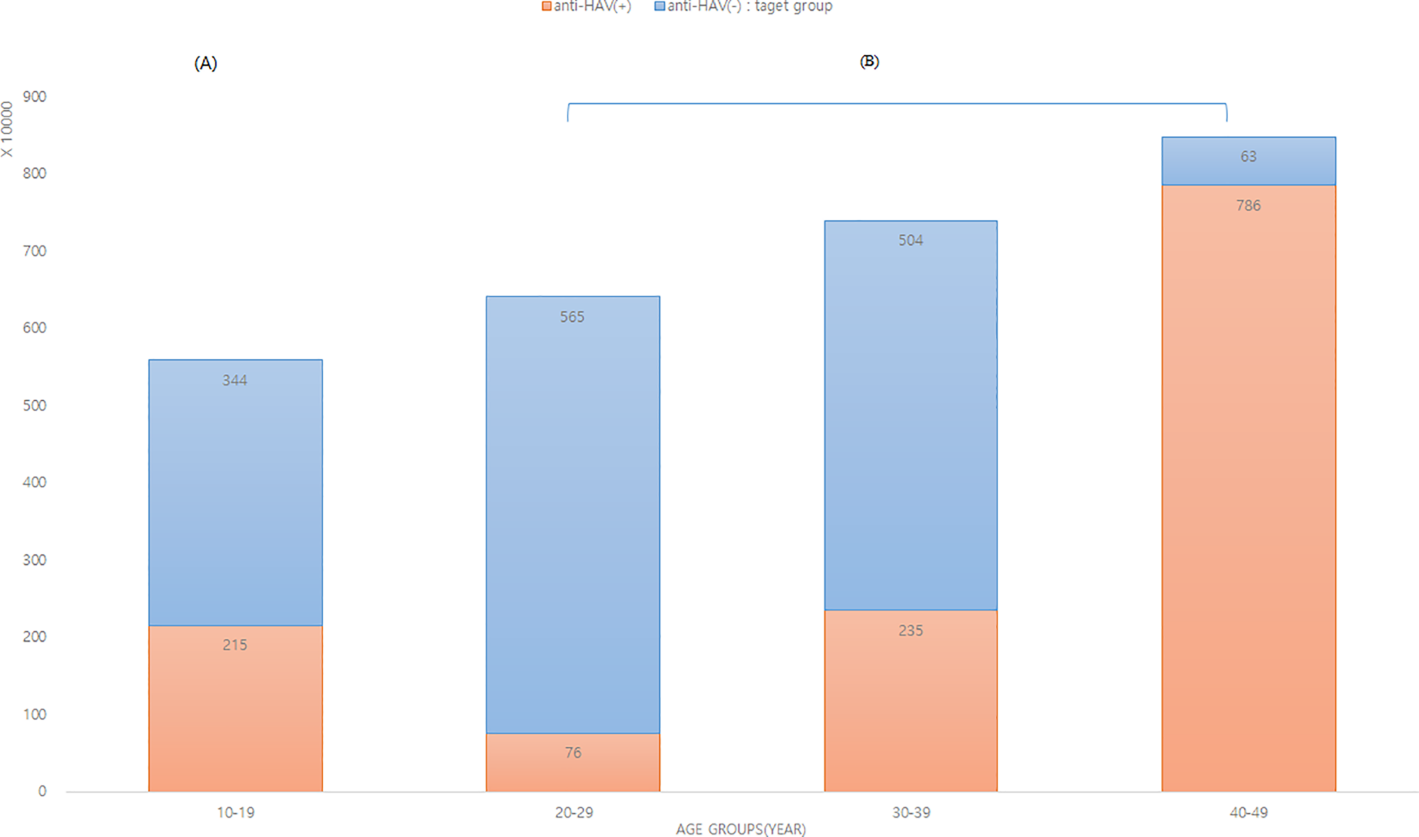
Target population of HAV vaccine in South Korea, bases on 2015 census. **(**A) Included in the national immunization recommendation of hepatitis A vaccine (B) In South Korea, the HAV vaccine for the children aged 12–26 months started in 1997 as a part of the National Childhood Vaccine Program. Children aged 10–19 years in 2015 were included in the program, but those aged 20 years and more were not included.

Cost can be a significant barrier due to limited public health resources to protect the health of adolescents and young adults by the catch-up vaccine program. Therefore, we calculated estimated target population of hepatitis A vaccine using the South Korean census at 2015 (Fig 3). There were approximately 14.7 million people without anti-HAV among those aged 10–49 years and only 340,000 among those aged 10–19 years. So theologically, the total cost of HAV vaccine will be 1.2 billion USD (only 0.27 among 10–19 years) to reach 80% of HAV vaccine coverage, with the the total amount of HAV vaccine being 2.4 billon doses.

The pre-vaccination serological testing can reduce the cost considering that anti-HAV testing costs approximately USD 10 and the price of 2 conventional doses of HAV vaccine in Korea is about USD 100. On the other hand, blind vaccination without serological testing among subgroups with very low positivity without history of infection or vaccination can be useful in specific situations. Therefore, we additionally we compared these two ways of vaccination. The blind vaccination strategy will cost only two doses (2A) of vaccine and two clinical visits (2B). Instead, the pre-vaccination testing strategy will cost serological testing (C) and one clinical visit, and according to the test results, two doses of vaccine (2A) and two clinical visits (2B), and the positive predicative rate of HAV (D) will be added. Otherwise, a second visit will be necessary to check the test result, which can be done within the same day as the first vaccination event. So, the expected total cost for the pre-testing strategy is C+B+(1-D)*(2A+2B) or C+2B+(1-D)*(2A+B) compared to 2A+2B for the blind vaccination strategy. Thus, in cases of D < (C+B)/(2A+2B) or < (C+B)/(2A+B), the blind strategy will be superior. In South Korea (C = 10 USD, B = 10 USD, A = 50 USD), the individuals with positive predictive rates of below 16.7% or 18.2% will benefit from reduced cost and lower number of visits.

### Limitation of the study

This study is subject to limitations. Anti-HAV tests results do not indicate whether the immunity is active or passive. Anti-HAV results in KNHANES are reported as a qualitative variable (positive or negative) with fixed cut-off points. This classification can underestimate the prevalence of anti-HAV positive individuals as the fixed cut-off points are usually selected to maximize specificity for diagnostic purposes. As KNHANES represents the non-institutionalized civilian population, there are some limitations to its generalizability. Finally, we combined children aged 10–14 and 15–19 years into a single group. While the positive rate of anti-HAV among children 10–14 years of age was relatively higher compared those aged 15–19 years, the exposure periods to hepatitis A environment between these two age groups were quite similar. Therefore, the difference in the positive rates of anti-HAV among those aged 10–14 years and 15–19 years is most likely due to differences in HAV vaccination.

### Final conclusion

In conclusion, the epidemiological transition of HAV seropositivity in South Korea is ongoing and a growing number of adolescents and young adults are susceptible to HAV infection. Since HAV vaccination from the National Childhood Vaccine Program in 1997 was a matter of choice, factors such as income and health behaviors contributed to differences in anti-HAV positivity. Since then, the campaign for childhood universal vaccination over 12 months have continued, and a government-funded nationwide routine vaccination program of children younger than 12 years started in 2015. Therefore, future studies investigating the impact of this new program are needed later on. We proposed a catch-up vaccination program for adolescents and young adults, with two doses of vaccine in not only South Korea but also in other countries experiencing similar epidemiological shifts. Catch-up vaccination seems to be cost-effective and affordable because this shift usually occurs in recently developed countries. Upon travelling to areas with high hepatitis A prevalence, young adults should consider being vaccinated for HAV. To provide reasonable government policy and evidence based clinical recommendation for hepatitis A vaccine, not only continuous nationwide survey of the HAV seroepidemiology but also study on the cost-effectiveness of catch-up vaccination are urgently required. In countries such as South Korea with ongoing epidemiological shifts, the need of hepatitis A vaccine is growing. Finally, in accordance with the recent increase in HAV vaccination, the possibility of vaccine shortage should be taken into account.

## Supporting information

S1 File Code book. (DOCX)

S2 File Raw data (all personal information removed). (DTA)
