# Supplementary material for: The effectiveness and limitation of the national childhood hepatitis A vaccination program in the Republic of Korea: Findings from the Korean National Health and Nutrition Examination Survey (KNHANES), 2015
Source: PLoS One. 2017 Dec 8;12(12):e0189210. doi: 10.1371/journal.pone.0189210 (PMC5722338; doi:10.1371/journal.pone.0189210)
Supplement: S1 File — (DOCX) [file pone.0189210.s001.docx]

DATA CODE BOOK for STATA

Variables and Labels

| Name of Variables | Labels |
| --- | --- |
| id | identification numbers |
| year | survey year |
| age | age(year) |
| sex | gender |
| ainc | home income per month |
| cfam | numbers of family |
| educ | education level |
| fedu | father's education level |
| medu | mother's education level |
| he_hepaa | positivity of hepatitis A antibody |
| bh9_11 | influenza vaccination |
